# Simultaneous Determination of 14 Phenolic Compounds in Grape Canes by HPLC-DAD-UV Using Wavelength Switching Detection

**DOI:** 10.3390/molecules181114241

**Published:** 2013-11-18

**Authors:** Ang Zhang, Li Wan, Cuiyun Wu, Yulin Fang, Guomin Han, Hua Li, Zhenwen Zhang, Hua Wang

**Affiliations:** 1 College of Enology, Northwest A&F University, Yangling 712100, Shaanxi, China; E-Mails: zhanganggrape@gmail.com (A.Z.); lilywanhp@126.com (L.W.); guomin_h@126.com (G.H.); lihuawine@nwsuaf.edu.cn (H.L.); zhangzhw60@nwsuaf.edu.cn (Z.Z.); wanghua@nwsuaf.edu.cn (H.W.); 2 Shaanxi Engineering Research Center for Viti-Viniculture, Yangling 712100, Shaanxi, China; 3 College of Plant Science, Tarim University, Alaer 843300, Xinjiang, China; E-Mail: wcyby@163.com

**Keywords:** HPLC-DAD-UV, wavelength switching, phenolic compounds, grape canes

## Abstract

The paper described a novel chromatographic method for the simultaneous determination of phenolic compounds such as gallic, protocatechuic, vanillic, caffeic, syringic, *p*-coumaric and salicylic acid, (+)-catechin, (‒)-epicatechin, rutin, morin, quercetin, coumarin and *trans*-resveratrol at their maximum absorbance wavelengths (MAW) employing reverse-phase high performance liquid chromatography combined with DAD and UV detection via detection wavelength switching. The method was based on MAW acquisition by DAD and quantification by UV. The separation process was performed on a Shim-Pack VP-ODS C_18_ column (250 mm × 4.6 mm, 5 μm) held at 30 °C, utilizing 3.0% acetic acid and acetonitrile as mobile phase at a flow rate of 0.8 mL/min in the gradient elution mode. The method was fully validated in terms of linearity (*r*^2^ > 0.9990, 10‒350 mg/L), precision (both intra-day and inter-day RSD < 4.22%), accuracy (97.31%‒104.66%), specificity, robustness (0.59% < RSD < 2.86%), limit of detection and quantification. The switching method significantly improved the sensitivities of most phenolics studied in comparison with the standard constant wavelength detection (280 nm). The proposed method has been successfully applied to the determination of 14 phenolic compounds in 89 varieties of one-year-old Chinese grape one-year-canes. Grape canes contain many phenolics, especially *trans*-resveratrol, (‒)-epicatechin, and (+)-catechin.

## 1. Introduction

Phenolic compounds have strong antioxidant activities associated with their abilities to scavenge free radicals, donate hydrogen, chelate metals, break radical chain reactions, and quench singlet oxygen *in vitro* and *in vivo* [1,2]. Among dietary antioxidants, phenolics are by far the most abundant compounds in most of the diets. Epidemiological studies have revealed the associations between the consumption of phenolic-rich foods and the prevention of oxidative stress-related diseases [3,4,5,6]. Concurrently, the synthetic antioxidants have restricted use in food as they are suspected to be carcinogenic [7]. People’s demand for natural products that can enhance and preserve health has never been greater with the enhancement of health consciousness.

The main solid wastes from vineyards and wineries, which include grape leaves, canes, pomace, and stems, are rich in high added-value natural antioxidants [8,9,10,11], especially in polyphenolic components. Compared with grape pomace and stems, people pay a little attention to grape canes with respect to the phenolic compound profile. The world total vineyard area reached approximately 7,636 mha in 2009 [12], with an approximate yearly grape cane yield of 1 t/ha [13], implying some 8,000,000 t of annual pruning waste production. These wastes represent a potentially important global source of natural antioxidants.

The content of phenolic compounds in biological samples can be determined by various analytical instrumental methods, such as gas chromatography [14], thin-layer chromatography [15], and capillary electrophoresis [16]. However, high performance liquid chromatography (HPLC) has proved to be the most appropriate owing to the structural similarity and diversity of phenolic compounds, allowing the analysis with sufficient precision, selectivity and within a reasonable time. HPLC typically hyphenated with ultraviolet visible (UV), photodiode array (DAD), mass spectrometry (MS), electrochemical (ED), fluorescence (FD), chemiluminescence (CL), refractive index (RI), and evaporative light scattering (ELSD) detectors has been the best method of choice for routine analysis of phenolic compounds in most hitherto published studies. However, in many cases, the disadvantages of some detectors were their limited analytical application because of baseline drift, limitations of detecting electrochemically inactive compounds (ED), complex pretreatment of non-fluorescent analytes (FD), fewer chemiluminescence reactions available, interference from excess use of some derivation reagents, incompatibility of the mobile phase with chemiluminescence reactions (CL) and low sensitivity (RI, ELSD) [17,18,19,20,21,22,23,24]. The critical decision for the analyst of which analytical technique to employ, is not only dependent on the expected composition of the sample and the designation of analytical expectations, but also certainly on the instrument availability.

Among all the detectors coupled with HPLC for the determination of phenolic compounds, MS is the most expensive and unusual one, especially for the labs with limited facilities. UV and DAD are the most useful and common ones in ordinary labs. Indeed a lot of previous papers existed which are about phenolic compound detection by HPLC via UV or DAD detection, but there is a universal phenomenon which is the non-uniform detection wavelengths adopted to determine the same or similar phenolic compounds. As we know, the optimum detection wavelengths for the determination of phenolics by HPLC-DAD-UV should be set for the sake of accurate quantitation, moreover, the MAW of phenolic compounds may differ due to their characteristic absorbance groups. To obtain the objective or true content of each phenolic compound in the matrix during the simultaneous determination, the proper detection wavelength should be set at the MAW for every compound. However, the wavelengths used in many literatures for phenolic compounds determination are not their MAW, even by DAD detection, which are usually a sort of compromise detection wavelength. A brief summary of UV and DAD detectors used in analysis of phenolic compounds with the emphasis to detection wavelength selection was displayed in Table 1.

**Table 1:** Detectors and wavelengths used in recent papers for phenolics detection.

Sample	Individual phenolics ^a^	Detector	Detection wavelength (nm)	Ref.
Grape & Wine	PCA, EC, PA, CA, GA, CAT, VA, SYA	UV-vis	280	[25]
Cumin organs	PCA, EC, PA, GA, QC, CAT, VA, SYA	UV-vis	280	[26]
Grape seeds	PCA, EC, GA, QC, CAT, RT, VA, SYA	UV-vis	280	[27]
Wine	RES	UV-vis	310	[28]
Jujube	GA, CA	UV-vis	280	[29]
Wine and tea	GA, PA, VA, CA, CAT, EC, SYA, QC, RT	UV-vis	280	[30]
	RT (Synthesized)	UV-vis	280	[31]
Mushroom	RT	UV-vis	300	[32]
Wine	EC, CA, QC, RES, CAT, RT	UV-vis	EC, CAT, RES-280; CA-320; QC, RT-360	[33]
Guava leaf	MR, GA, QC, CAT	UV-vis	280	[34]
Grape waste	CA, GA, QC, RES, CAT, RT, SYA	UV-vis	280	[35]
Wine	QC, RT, MR	UV-vis	360	[36]
Cheonggukjang	CA, EC, PA, MR, GA, CAT, VA	UV-vis	280	[37]
Plant material	PHA, VA, CA, SYA, PCA	UV-vis	254	[38]
Wine	PCA, EC, PA, CA, GA, QC, RES, CAT, VA	DAD	280	[39]
Knotweed	CAT, EC, RES	DAD	RES-315; CAT, EC-220	[40]
Grape cane	RES	DAD	320	[10]
Mescal	SYA	DAD	260	[41]
Tea	GA, PA, VA, CA, CAT, EC, RT, QC.	DAD	280	[42]
Peanut skin	RES	DAD	280	[43]
Ma-mao juice	EC, QC, RES, CAT, RT	DAD	254	[44]
Guava leaf	GA, CAT, QC	DAD	280	[45]
Beverage	CAT, EC, QC	DAD	QC-360; CAT, EC-230	[46]
Grape seed and skin	EC, GA, CAT, VA, SYA	DAD	280	[47]
Grape product	GA, CAT, EC, RES, CA, PCA, QC	DAD	GA, CAT, EC-280; RES, CA, PCA-320; QC-360	[48]

^a^ CA caffeic acid; CAT (+)-catechin; CR coumarin; EC (‒)-epicatechin; GA gallic acid; MR morin; PA protocatechuic acid; PCA *p*-coumaric acid; QC: quercetin; RES *trans*-resveratrol; RT rutin; SLA salicylic acid; SYA syringic acid; VA vanillic acid.

Ten of or tens of nanometers of detection wavelength differences usually happen during the determination of the same compounds, this may result in non-detected or false values for some compounds in trace detection and lead discrepancies and incomparability of the results, especially for the same analytes analyzed by different researchers. A DAD detector could simultaneously scan samples at multiple wavelengths and provide the information of the special spectral characteristics for identification of compounds, but its sensitivity is lower than that of UV detectors for quantitation [49]. To overcome these pitfalls, we proposed a wavelength-switching method by HPLC coupled with a DAD detector for MAW acquisition and a UV detector for quantitation.

In this work, an optimized and exhaustively validated method for the simultaneous analysis of gallic acid, protocatechuic acid, (+)-catechin, vanillic acid, caffeic acid, syringic acid, (‒)-epicatechin, *p*-coumaric acid, rutin, salicylic acid, coumarin, *trans*-resveratrol, morin, and quercetin in 89 varieties of Chinese grape canes was developed using HPLC-DAD-UV with wavelength switching detection. In addition, the sensitivities of UV and DAD were determined under both constant and wavelength switching detection, and fine-tuning of wavelength of the UV detector was investigated to indicate the differences in results of MAW and common wavelengths detection.

## 2. Results and Discussion

### 2.1. Optimization of Chromatographic Conditions

**Figure 1:**
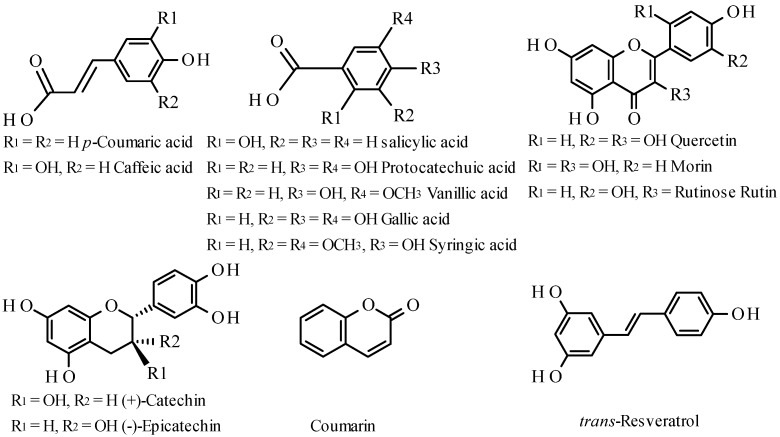
Chemical structures of phenolic compounds.

The aim of this study was to establish a sequential injection procedure, which has the ability to provide suitable separation conditions for the determination of gallic acid, protocatechuic acid, (+)-catechin, vanillic acid, caffeic acid, syringic acid, (‒)-epicatechin, *p*-coumaric acid, rutin, salicylic acid, coumarin, *trans*-resveratrol, morin, and quercetin (Figure 1), and to demonstrate the feasibility of applying this method to real samples. It is well known that the elution order of phenolic compounds in RP-HPLC is closely related to their polarity, with the most polar ones eluting first, followed the less polar ones. Once the analyte types are identified, the parameters affecting HPLC retention performance such as sample solvents, mobile phase composition, column temperature, and flow rate should be optimized.

Eight varieties of mobile phases (methanol/water, acetonitrile/water, methanol/1%, 2%, 3% acetic acid, and acetontrile/1%, 2%, 3% acetic acid), three column temperatures (28, 30, and 32 °C), and three flow rates (0.6, 0.8, and 1.0 mL/min) were tested to screen optimal chromatographic conditions. Although methanol and acetonitrile as mobile phase had similar strength, peaks for phenolic compounds eluted by methanol were broader than those by acetonitrile in this study. Some asymmetrical peaks (catechin, syringic acid, and morin) and very late eluting peaks (morin and quercetin) were also observed in methanol elution compared to acetonitrile. This might be attributed to the higher dipole moment of acetonitrile, with the resulting stronger analyte-mobile phase dispersive interaction (donor-acceptor interactions). The sharp peaks and shorter run times observed when using acetonitrile were similar to the results reported by other authors [50,51]. Peaks of vanillic acid and caffeic acid were found to be difficult to separate in a chromatogram both with methanol and acetonitrile due to their similar retention properties, with resolution factors of 1.28 and 1.43, respectively. The use of 3% acetic acid solution as a mobile phase additive achieved good separation and resolution of all the phenolic compounds of interest in this study. The optimized flow rate and column temperature were 0.8 mL/min and 30 °C, respectively, for the purposes of shortening analysis times and improving peak shape after several tests.

Phenolic compounds absorb well in the UV range with different absorptive intensity and response times due to their respective characteristic structures, therefore, choosing suitable detection wavelengths for each analyte should be seriously considered and no single one is sufficient for the simultaneous determination in extracts of various plant materials. MAWs of 14 phenolic compounds scanned by the DAD detector were listed in Table 2. Although phenolic compounds have more than one absorption peaks in their DAD spectra, except for gallic acid, (+)-catechin, syringic acid, (‒)-epicatechin, *p*-coumaric acid, and *trans*-resveratrol, the more intense ones were chosen as their detection wavelengths. There were just three phenolic compounds with the same maximum absorbance at 280 nm. Apparently, detection at 280 nm, the most general detection wavelength used for the simultaneous determination of different phenolic compounds, was insufficient.

**Table 2:** Data of MAW, retention, response, and switching times for phenolic compounds.

Name	Retention time ± SD	MAW (nm)	Response time, duration (min)	Switching time, duration (min)
Gallic acid	5.883 ± 0.014	271	5.513–6.243, 0.730	5.463–6.293, 0.830
Protocatechuic acid	8.932 ± 0.015	260	8.707–9.157, 0.449	8.657–9.207, 0.549
(+)-Catechin	12.705 ± 0.019	280	12.205–13.125, 0.920	12.155–13.175, 1.020
Vanillic acid	18.637 ± 0.018	260	18.324–18.997, 0.673	18.274–19.047, 0.773
Caffeic acid	20.574 ± 0.020	324	20.129–21.025, 0.896	20.079–21.075, 0.996
Syringic acid	31.683 ± 0.019	275	31.174–32.184, 1.010	31.124–32.234, 1.110
(‒)-Epicatechin	33.712 ± 0.011	280	33.28–34.133, 0.853	33.230–34.183, 0.953
*p*-Coumaric acid	37.486 ± 0.017	309	37.143–37.835, 0.692	37.093–37.885, 0.792
Rutin	41.058 ± 0.019	255	40.882–41.234, 0.352	40.832–41.284, 0.452
Salicylic acid	44.927 ± 0.016	304	44.502–45.353, 0.851	44.452–45.403, 0.951
Coumarin	49.384 ± 0.018	280	48.881–49.886, 1.005	48.831–49.936, 1.105
*trans*-Resveratrol	53.115 ± 0.015	306	52.610–53.624, 1.014	52.560–53.674, 1.114
Morin	55.867 ± 0.015	256	55.251–56.489, 1.238	55.201–56.539, 1.338
Quercetin	62.342 ± 0.020	374	61.839–62.845, 1.006	61.789–62.895, 1.106

**Figure 2:**
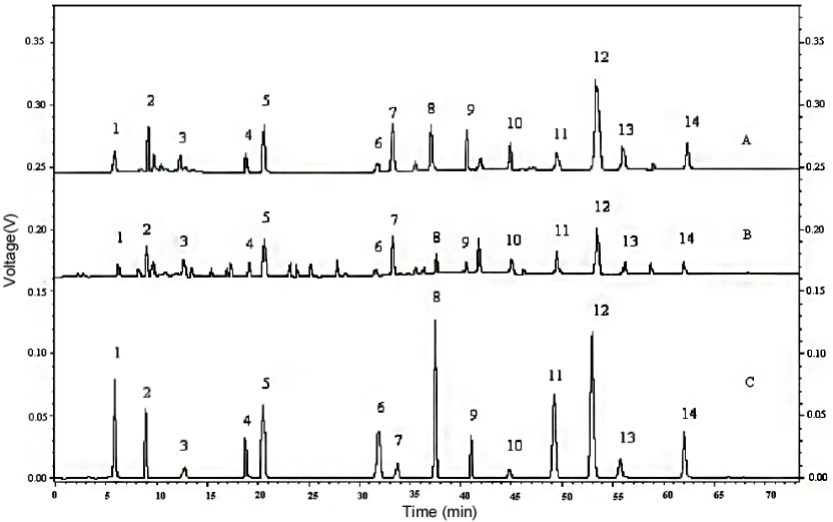
Typical HPLC chromatograms **A**: Cabernet Sauvignon extract at switching wavelength; **B**: Cabernet Sauvignon extract at 280 nm; **C**: Standard compounds at switching wavelength. Peaks: 1‒Gallic acid; 2‒Protocatechuic acid; 3‒(+)-Catechin; 4‒Vanillic acid; 5‒Caffeic acid; 6‒Syringic acid; 7‒(‒)-Epicatechin; 8‒*p*-Coumaric acid; 9‒Rutin; 10‒Salicylic acid; 11‒Coumarin; 12‒*trans*-Resveratrol; 13‒Morin; 14‒Quercetin.

The time points for wavelength switching for each compound were set based on their retention and response times acquired from linearity experiments, as can be seen in Table 2. The retention time in 18 continuous analysis indicated excellent repeatability expressed as RSD (<0.27%). The Shimadzu HPLC allows random wavelength switching between 190 and 400 nm, the detector’s response and stabilization time is less than 0.1 s (data from Shimadzu Technique). In this experiment, the detection wavelength was switched within 0.05 min before peak signal starting and after its stopping, which implied that each phenolic compound could be detected at its MAW. The wavelength at other time periods was set to 360 nm, which minimized the interferences from solvents and impurities. The representative chromatograms of the phenolic standards and sample separation are shown in Figure 2. Wavelength-switching detection gave a more effective chromatogram with less impurity peaks than did the constant wavelength (280 nm) one.

### 2.2. Fine-Tuning of Detection Wavelength

**Figure 3:**
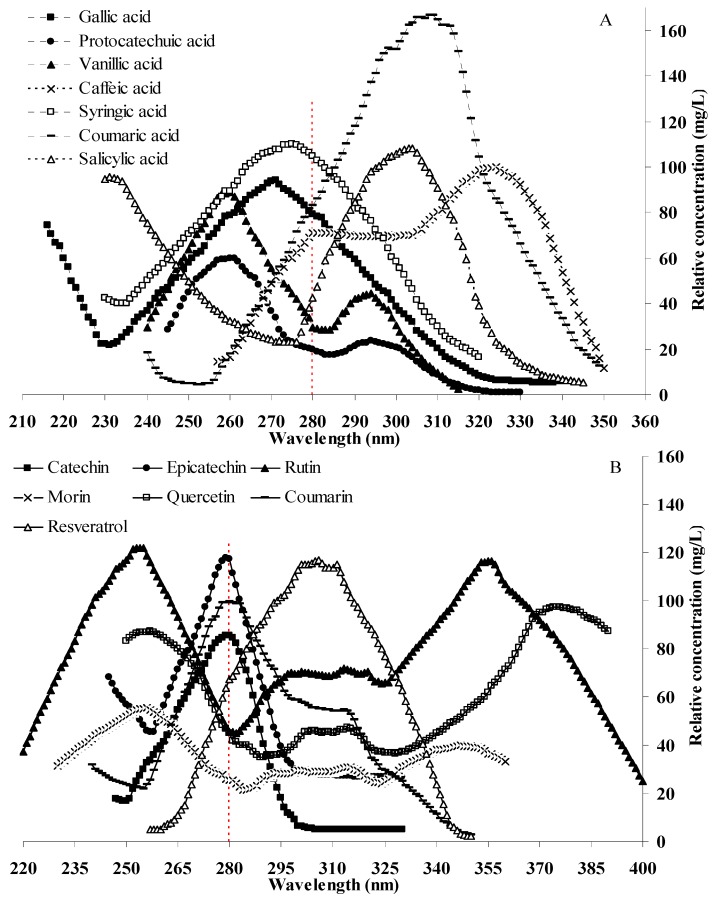
Relative concentrations of phenolic compounds under different detection wavelengths (**A**) phenolic acids; (**B**) flavonoids, coumarin, and *trans*-resveratrol).

The wavelength fine-tuning data manifested the relationship between the relative concentrations and the detection wavelengths for the same concentrated standard solution. The results are shown in Figure 3. It can be seen that the relative concentrations of different compounds varied with wavelengths dramatically. Compared with detection at 280 nm, the relative concentrations of protocatechuic acid and vanillic acid were almost three times lower than those at their MAW, and gallic acid, caffeic acid, *p*-coumaric acid, and salicylic acid were about 1.2, 1.5, 2, and 2.5 times, respectively, as can be seen in Figure 3A. The relative concentrations of rutin, morin, and quercetin determined at their MAW were almost 2.7, 2.2, and 2.2 times higher than those at 280 nm, and *trans*-resveratrol at 280 nm was 1.7 times lower in comparison with its MAW (Figure 3B). The detection wavelengths of 14 phenolic compounds with the highest relative concentrations were in conformity with the MAW given by the DAD detector.

### 2.3. Validation of the Method

The analytical method, proposed for the determination of 14 phenolic compounds in 1-yr-old grape canes by HPLC-DAD-UV with wavelength switching detection, was validated in terms of linearity, range, LOD, LOQ, specificity, accuracy, precision and robustness.

Method linearity is the ability to produce the results that are directly proportional to the concentrations of analytes in the sample within a given range. In the present study, linearity was evaluated by plotting peak area (A) *versus* analyte concentration (C, mg/L) to construct the calibration curves. Regression equations of 14 phenolic compounds listed in Table 3 were linear in the investigated range, with the lowest correlation coefficient ≥ 0.9990, indicating excellent linearity.

A signal-to-noise ratio of approximately 3 is generally considered to be acceptable for estimating the detection limit. The quantitation limit with a typical signal-to-noise ratio of 10 is the lowest concentration of the analyte that can be quantified with acceptable precision and accuracy. LOD and LOQ were separately analyzed by diluting the standard solution. The LOD and LOQ, both for UV-vis and DAD detectors under constant (280 nm) and switching wavelength detection, were studied while keeping all other chromatographic conditions the same, details are given in Table 3. Regarding sensitivity, the UV-Vis detector with wavelength switching had the highest sensitivity for determination of phenolic compounds in comparison with that achieved by both UV-Vis and DAD detector with constant detection or DAD with wavelength switching.

**Table 3:** Results of calibration and sensitivity, including LOD and LOQ of UV and DAD detectors under different detection wavelength modes (mg/L).

Name ^a^	Linear equation ^b^	Corr. coeff. (*r*^2^)	UV detector				DAD detector			
			Constant (280 nm)		Switching		Constant (280 nm)		Switching	
			LOD	LOQ	LOD	LOQ	LOD	LOQ	LOD	LOQ
GA	C = 46051(±385)A − 209260(±3821)	0.9993	0.032	0.098	0.021	0.062	0.064	0.188	0.043	0.119
PA	C = 27162(±321)A − 74702(±275)	0.9993	0.044	0.125	0.015	0.043	0.134	0.387	0.048	0.151
CAT	C = 11372(±199)A − 49365(±328)	0.9991	0.026	0.073	0.026	0.073	0.120	0.290	0.120	0.290
VA	C = 6648(±45)A + 1235(±41)	0.9991	0.068	0.182	0.033	0.095	0.098	0.285	0.062	0.179
CA	C = 85705(±612)A − 283259(±4372)	0.9996	0.037	0.110	0.025	0.077	0.126	0.368	0.099	0.302
SYA	C = 49634(±654)A − 106819(±1422)	0.9993	0.013	0.038	0.009	0.026	0.034	0.097	0.040	0.116
EC	C = 10877(±162)A − 73865(±705)	0.9992	0.062	0.358	0.062	0.358	0.139	0.508	0.139	0.508
PCA	C = 66221(±524)A − 138690(±2312)	0.9997	0.019	0.058	0.013	0.037	0.047	0.145	0.030	0.085
RT	C = 19525(±327)A − 29519(±342)	0.9990	0.065	0.182	0.022	0.064	0.108	0.331	0.087	0.273
SLA	C = 9282(±76)A − 46686(±366)	0.9995	0.121	0.356	0.052	0.148	0.235	0.698	0.104	0.317
CR	C = 65717(±678)A − 44484(±621)	0.9996	0.034	0.133	0.034	0.133	0.136	0.405	0.136	0.405
RES	C = 94435(±628)A + 250679(±2313)	0.9997	0.007	0.023	0.003	0.008	0.067	0.207	0.032	0.092
MR	C = 12833(±465)A + 73026(±665)	0.9991	0.037	0.112	0.023	0.060	0.122	0.371	0.081	0.237
QC	C = 31436(±973)A − 77114(±768)	0.9998	0.041	0.125	0.017	0.053	0.142	0.422	0.076	0.232

^a^ GA: Gallic acid; PA: Protocatechuic acid; CAT: (+)-Catechin; VA: Vanillic acid; CA: Caffeic acid; SYA: Syringic acid; EC: (‒)-Epicatechin; PCA: *p*-Coumaric acid; RT: Rutin; SLA: Salicylic acid; CR: Coumarin; RES: *trans*-Resveratrol; MR: Morin; QC: Quercetin. ^b^ Linear ranges of all compounds were 10-350 mg/L.

**Figure 4:**
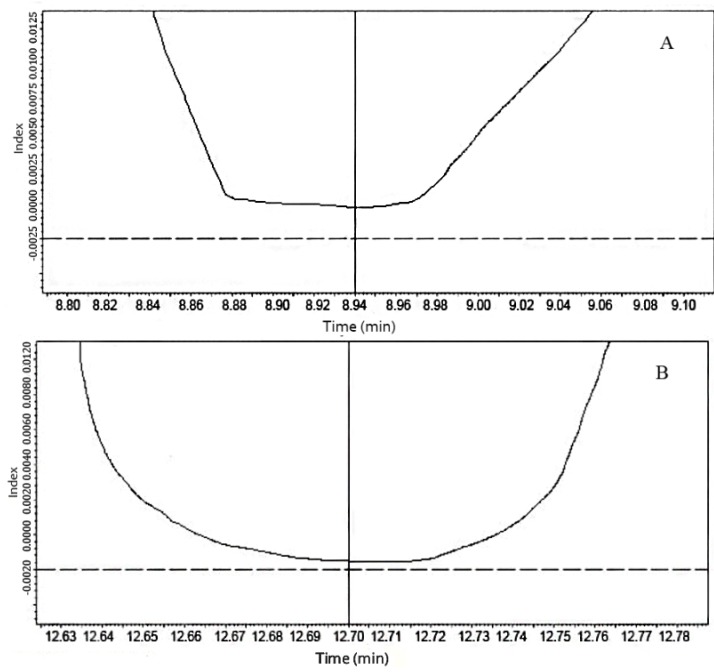
Typical peak purity curves (**A**) Protocatechuic acid; (**B**) Catechin.

Method specificity ensures that the signal measured comes from the analyte of interest, with no interference from any potential sample components. Chromatographic identification of phenolic compounds was based on comparing retention times and ultraviolet absorption spectrums. The representative chromatograms of the standard mixture solution and Cabernet Sauvignon extract separation are depicted in Figure 2. In the present case, some impurities found in samples, could be some interferences because of the complexity of the crude extracts injected directly. The resolution factors calculated by the Shimadzu software between (+)-catechin and nearby interference peaks in the sample were 1.6 and 1.7, respectively (data not shown). Moreover, in order to verify the specificity, peak purity was evaluated by the PDA detector for the phenolics in samples and the standard solution. Two typical peak purity curves of the samples are shown in Figure 4 for protocatechuic acid and (+)-catechin, and no impurities were observed. Thus, the method was confirmed to be specific for each phenolic compound of interest.

Evaluation of method accuracy resorted to the standard addition technique. Extracts of Cabernet Sauvignon were used as substrates for recovery test, as mentioned in Section 2.7, adding known amounts of phenolic reference compounds to the extracts with known amounts, to obtain three addition levels (50, 150, and 250 mg, three replicates each). Each set of samples was repeated three times and the average recovery rate of each compound was as laid out in Table 4, ranging from 97.31% to 104.66%, indicating that the method was accurate.

Precision was determined as both repeatability and intermediate precision, in accordance with ICH recommendations. The spiked extracts used in accuracy analysis were also served in precision study, the repeatability expressed as a relative standard deviation (RSD, %) was determined using six continuous injections of three concentrations and analysed on the same day, the intermediate precision was evaluated for 14 working days by injecting the same test solution three times a day. The precision data is tabulated in Table 4. In both situations, all the RSD values were below 4.22%, demonstrating that the method was precise.

**Table 4:** Validation results for accuracy (*n*= 3), precision (intra-day, *n* = 6; inter-day, *n* = 14), and robustness (*n*= 3).

Name ^a^	Control (mg)	Added (mg)	Found ± SD (mg)	Recovery (%)	Precision RSD (%)		Robustness RSD (%)					
					Intra-day	Inter-day	Flow rate (mL/min)		AcOH conc. (%, v/v)		Column temp. (°C)	
							0.75	0.85	2.95	3.05	29	31
GA	43.86	50	91.34 ± 2.26	97.31	2.67	2.80	1.43	2.31	2.55	2.13	0.84	0.65
		150	195.59 ± 7.48	100.89	3.72	3.86						
		250	292.86 ± 10.88	99.66	3.35	3.13						
PA	140.57	50	192.11 ± 5.57	100.81	2.72	3.33	1.67	1.73	2.35	2.86	1.29	0.98
		150	297.21 ± 8.72	102.32	2.65	3.01						
		250	395.14 ± 13.46	101.17	3.05	3.76						
CAT	733.77	50	799.92 ± 23.83	102.06	1.62	3.80	1.53	1.88	2.06	2.17	0.58	1.05
		150	885.98 ± 25.49	100.25	2.80	3.90						
		250	984.56 ± 36.48	100.08	3.79	3.92						
VA	46.84	50	100.09 ± 2.46	103.36	2.71	3.57	1.48	1.62	1.76	1.89	0.86	0.99
		150	195.86 ± 5.22	99.50	2.82	3.10						
		250	297.70 ± 9.87	100.29	2.96	3.13						
CA	44.56	50	95.28 ± 2.51	100.76	2.54	3.74	1.32	1.44	2.73	2.85	0.67	0.59
		150	195.92 ± 6.14	100.70	3.32	3.65						
		250	296.71 ± 11.32	100.73	3.42	3.69						
SYA	113.63	50	160.54 ± 3.78	98.11	2.26	2.51	1.76	1.85	2.65	1.87	1.12	1.06
		150	272.96 ± 6.27	103.24	2.43	2.76						
		250	367.48 ± 10.78	101.06	3.11	3.22						
EC	545.71	50	600.18 ± 14.64	100.75	2.54	3.10	1.67	1.79	2.21	1.99	0.88	1.10
		150	722.84 ± 21.73	103.90	3.14	3.29						
		250	803.27 ± 34.32	100.95	3.74	4.01						
PCA	77.55	50	133.49 ± 3.39	104.66	2.09	2.76	1.58	1.62	2.71	2.90	0.84	0.93
		150	229.53 ± 6.67	100.87	3.02	2.93						
		250	327.26 ± 10.43	99.91	3.26	3.44						
RT	92.29	50	142.55 ± 4.36	100.18	1.79	2.67	1.78	1.75	2.65	2.68	0.79	0.89
		150	244.45 ± 6.03	100.89	2.40	2.54						
		250	344.07 ± 7.51	100.52	2.41	2.84						
SLA	179.62	50	229.37 ± 6.49	99.89	2.58	2.98	1.46	1.42	2.47	2.80	1.05	1.32
		150	336.64 ± 10.54	102.13	2.94	3.26						
		250	431.94 ± 13.84	100.54	2.93	3.35						
CR	25.33	50	75.85 ± 2.13	100.69	3.74	4.22	1.87	1.73	2.12	2.34	0.59	0.66
		150	171.22 ± 5.38	97.65	2.84	3.15						
		250	277.59 ± 8.37	100.82	3.07	3.31						
RES	1048.7	50	1125.44 ± 35.82	102.43	2.91	3.25	1.59	1.60	2.83	2.76	1.07	0.74
		150	1191.31 ± 39.27	99.38	3.16	3.21						
		250	1318.09 ± 41.22	101.49	2.95	3.24						
MR	192.68	50	252.90 ± 3.72	104.21	1.70	2.92	1.55	1.49	2.37	2.51	0.82	0.77
		150	344.29 ± 8.28	100.47	2.56	2.56						
		250	436.31 ± 14.28	98.56	3.20	3.20						
QC	87.85	50	137.63 ± 3.37	99.84	3.08	3.16	1.69	1.36	2.54	2.87	1.21	1.14
		150	245.65 ± 7.99	103.28	3.05	3.43						
		250	349.91 ± 10.43	103.57	3.26	3.53						

^a^ GA: Gallic acid; PA: Protocatechuic acid; CAT: (+)-Catechin; VA: Vanillic acid; CA: Caffeic acid; SYA: Syringic acid; EC: (‒)-Epicatechin; PCA: *p*-Coumaric acid; RT: Rutin; SLA: Salicylic acid; CR: Coumarin; RES: *trans*-Resveratrol; MR: Morin; QC: Quercetin.

Robustness testing is performed to evaluate the influence of small, but deliberate, variations in method parameters. The parameters chosen in this study were the flow rate (±0.05 mL/min), acetic acid concentration (±0.05%, v/v) and column temperature (±1 °C). Three injections of the mixed standard solution were carried out for each variation. The degree of reproducibility of peak area for each compound expressed as RSD was calculated. The results are illustrated in Table 4, an RSD of 0.59%–2.86% was obtained for these parameters. Hence, the analytical method could be considered to be robust.

### 2.4. Application

In order to validate the utility of the proposed method in real samples, gallic acid, protocatechuic acid, vanillic acid, caffeic acid, syringic acid, *p*-coumaric acid, salicylic acid, (+)-catechin, (‒)-epicatechin, rutin, morin, quercetin, coumarin, and *trans*-resveratrol in 89 varieties of grape canes were detected by the method. All the sample extracts prepared according to the procedure described in Section 2.3 were injected into the instrument with an injection amount of 10 µL (*n* = 3) and the peaks in the chromatograms obtained were identified by comparison of retention times, UV spectrums, and the increase of peak area after the addition of the corresponding phenolics. The typical chromatogram is shown in Figure 2A, and the average contents of the compounds are presented in Table S1. The results demonstrate that grape cane contains a great quantity of phenolic compounds and is an important source of natural antioxidants, especially for *trans*-resveratrol, (‒)-epicatechin, and (+)-catechin.

## 3. Experimental

### 3.1. Chemicals

HPLC grade methanol, acetonitrile, and analytical grade acetic acid were purchased from Tianjin Kermel Chemical Reagent Co. Ltd. (Tianjin, China). Water was purified by Milli-Q system (Millipore, Bedford, MA, USA). All the phenolic compounds were supplied from Sigma-Aldrich (Shanghai, China) and their purities were all over 97%.

### 3.2. Preparation of Plant Materials

The ideal 1-yr-old canes used in this work from 89 kinds of grapes were moderately vigorous (about 0.8‒1.0 cm diameter) and were collected from a commercial vineyard located in Shandong Province during the 2008 pruning practice. The 1-yr-old canes were frozen in liquid nitrogen, ground through a 0.5 mm sieve using an electrical grinder (final particle size <0.5 mm), vacuumized in labeled plastic bags to cut off air, and then stored at −20 °C in a freezer until extraction.

### 3.3. Extraction Process

The ground grape cane (5 g fresh weight) was weighed into a 50 mL centrifuge tube and extracted with 40 mL acidified methanol solution (1 N HCl/methanol/water, 1/80/19, v/v/v), and extraction was performed under continuous stirring (600 rpm) at 20 °C for 1 h by an external water bath. After extraction, the extracts were centrifuged at 8,000 g for 15 min at 4 °C using a Sorvall RC-5C Plus centrifuge (Kendro Laboratory Products, Newton, CT, USA). The extraction procedure was repeated three times. All the supernatants were combined in a 250 mL round-bottom flask and concentrated in a Büchi RE-111 rotary vacuum evaporator with a 35 °C water bath (Flawill, Switzerland) to a volume of 10 mL. The final concentrate solution was filtered through a 0.22 µm nylon micro-membrane and stored at −40 °C until analysis.

### 3.4. Preparation of Standard Solution

A mixed stock standard solution of 1 mg/mL was prepared by accurately weighing 25 mg of 14 phenolic compounds into 25 mL volumetric flask and making up to volume with HPLC grade methanol. Different working solutions were obtained from the stock solution by appropriate dilution in methanol for calibration curves and determinations of the detection and quantitation limit of the method. All the solutions were kept in dark place at −40 °C prior to injection.

### 3.5. HPLC-DAD-UV Analyses

The HPLC analyses were conducted on a Shimadzu liquid chromatograph system (Shimadzu Corp, Kyoto, Japan) equipped with a quaternary pump, a vacuum degasser, an autosampler, a PDA detector, a tunable UV-vis detector, and a Shim-Pack VP-ODS C_18_ column (250 mm × 4.6 mm, 5 μm). Results were acquired and processed by the Shimadzu Workstation CLASS-VP 6.12 software (Shimadzu Corp).

The DAD detector was applied to scan the phenolic compounds of interest to ascertain their MAW and acquire other spectral information within a range of 200 to 400 nm. The variable UV-Vis detector was conducted at the MAW of each phenolic compound for quantitative purpose with external standard. A gradient solvent system was employed with solvent A being water-acetic acid (97:3, v/v) and solvent B being acetonitrile. The elution profile had the following proportions (v/v) of solvent B: 0.00–5.00 min, 0%–8.5%; 5.00–16.50 min, 8.5%–2.0%; 16.50–35.00 min, 2.0%–18%; 35.00–50.00 min, 18%–20%; 50.00–65.00 min, 20%–30%; 65.00–70.00 min, 0%–30%. The following wavelength-switching program was employed: 5.463–6.293 min, 271 nm; 8.657–9.207 min, 260 nm; 12.155–13.175 min, 280 nm; 18.274–19.047 min, 260 nm; 20.079–21.075 min, 324 nm; 31.124–32.234 min, 275 nm; 33.230–34.183 min, 280 nm; 37.093–37.885 min, 309 nm; 40.832–41.284 min, 255 nm; 44.452–45.403 min, 304 nm; 48.831–49.936 min, 280; 52.560–53.674 min, 306 nm; 55.201–56.539 min, 256 nm; 61.789–62.895 min, 374 nm; 360 nm was for other time. The column held at 30 °C was flushed with a flow rate of 0.8 mL/min. Chromatographic identification and confirmation of phenolic compounds were based on comparing retention times with authentic standards and on-line ultraviolet absorption spectrum data. All the prepared solutions were filtered through 0.22 µm membranes, and the mobile phase was degassed before injection on to HPLC.

### 3.6. Fine-Tuning Analysis of Detection Wavelength

Fine-tuning of wavelength detection was carried out by the determination of a mixed standard solution with the known concentration in the UV-vis detector under the chromatographic condition described in 2.5 at the wavelength changing from 210 to 400 nm, with 1 nm interval. The raw data recorded was the peak area of each compound in the chromatograms; the relative concentrations at different wavelengths were calculated from the calibration curves.

### 3.7. Method Validation Procedure

The method proposed in this study was validated as per ICH guidelines by the determination of the following parameters: linearity, range, precision, accuracy, specificity, robustness (ICH Q2A, ICQ Q2B) [52], limit of detection (LOD) and limit of quantitation (LOQ).

Linearity of the method was established by automatic injections of the standard mixture solutions at six calibration levels in three replicates from low to high concentrations; retention time and response time (duration of signal response in detector) of each compound were extracted by the Shimadzu software for the studying of wavelength switching. Specificity of the method was evaluated by comparing the chromatograms both of standard solutions and the samples through the peak identification and peak purity assessment. Method precision was tested by determining the intra-day precision (repeatability) and the inter-day precision (intermediate precision), both expressed as RSD (%). The accuracy of the method was assessed by spiking phenolic compounds at three levels to samples and was expressed in terms of the average recovery. Method robustness was determined by making slight changes in the chromatographic conditions, such as flow rate, column temperature, and mobile phase additive concentration. LOD and LOQ of both DAD and UV-Vis detector were separately determined by diluting the standard solution.

## 4. Conclusions

Research on phenolic compounds is of current interest since they have important biological and pharmacological properties. HPLC, with various detection possibilities, or their combinations, has been a preferred technique for routine analysis of phenolics. In this paper, a novel HPLC-DAD-UV method using wavelength switching detection has been established for the simultaneous determination of 14 phenolic compounds in crude grape cane extracts without pre-treatment. Moreover, the proposed procedure, characterized by good sensitivity, linearity, precision, accuracy, and robustness, enabling each compound determined at their MAWs to obtain the real contents to the greatest extent, has an application potential to other analytes and can be suitable for routine laboratories without advanced facilities. In addition, grape canes rich in natural antioxidants should receive more attention.

## Supplementary Materials

Supplementary materials can be accessed at: http://www.mdpi.com/1420-3049/18/11/14241/s1.
