# Supplementary material for: Simultaneous Determination of 14 Phenolic Compounds in Grape Canes by HPLC-DAD-UV Using Wavelength Switching Detection
Source: Molecules. 2013 Nov 18;18(11):14241–57. doi: 10.3390/molecules181114241 (PMC6269983; doi:10.3390/molecules181114241)
Supplement: Supplementary file 1 [file molecules-18-14241-s001.pdf]

Supplementary Materials

Table S1. Contents of individual phenolic compounds in 89 varieties of one-year-old grape canes (mg kg^{-1} of fresh cane powder).

Variety (*V. vinifera L*)	Gallic acid	Protocatechuic acid	Catechin	Vanillic acid	Caffeic acid	Syringic acid	Epicatechin	*p*-Coumaric acid	Rutin	Salicylic acid	Coumarin	*trans*-Resveratrol	Morin	Quercetin
8804	48.29 ± 2.43	143.93 ± 3.42	593.31 ± 18.40	47.56 ± 1.45	45.58 ± 2.35	98.84 ± 3.28	543.34 ± 13.84	81.43 ± 3.47	72.52 ± 1.85	181.65 ± 1.87	35.55 ± 0.57	789.93 ± 35.67	164.56 ± 5.39	74.51 ± 2.49
Alicante Bouschet	26.22 ± 0.41	154.53 ± 2.33	548.08 ± 15.79	55.98 ± 2.12	58.22 ± 2.87	102.07 ± 6.77	512.63 ± 24.58	76.63 ± 1.79	85.73 ± 1.32	170.54 ± 2.42	21.76 ± 0.93	920.97 ± 33.28	180.37 ± 4.30	85.76 ± 2.06
Aligoté	17.65 ± 0.62	117.31 ± 2.35	436.63 ± 16.43	23.72 ± 0.76	45.82 ± 0.93	85.53 ± 1.76	402.54 ± 31.44	94.32 ± 2.92	64.53 ± 2.43	182.66 ± 4.92	49.58 ± 1.05	837.65 ± 43.50	132.52 ± 5.49	67.88 ± 3.39
Angelina	15.3 ± 0.26	121.34 ± 1.78	557.39 ± 15.65	35.73 ± 2.09	36.48 ± 0.68	87.36 ± 3.78	415.37 ± 23.42	71.84 ± 2.28	66.77 ± 0.93	171.05 ± 6.45	22.74 ± 1.47	923.04 ± 38.49	184.49 ± 5.74	68.57 ± 3.67
Augusta	25.76 ± 0.87	104.58 ± 4.32	389.29 ± 18.58	19.74 ± 0.48	30.94 ± 1.32	84.29 ± 2.48	478.45 ± 23.12	85.28 ± 3.75	59.18 ± 1.38	193.49 ± 7.33	44.29 ± 0.48	789.38 ± 27.38	149.48 ± 3.29	56.38 ± 2.67
Autumn Black	21.88 ± 0.17	124.82 ± 2.47	611.94 ± 23.43	39.84 ± 1.37	49.57 ± 0.94	73.63 ± 3.78	521.24 ± 23.41	74.52 ± 1.78	62.88 ± 0.74	168.36 ± 5.83	22.77 ± 0.82	906.73 ± 24.52	193.82 ± 4.53	69.61 ± 1.45
Autumn Royal	24.76 ± 1.21	110.43 ± 5.29	432.48 ± 15.98	23.54 ± 1.13	38.25 ± 2.48	74.29 ± 2.18	468.65 ± 23.74	104.38 ± 3.24	62.68 ± 1.45	174.39 ± 5.38	38.49 ± 0.45	1022.38 ± 43.18	155.29 ± 5.58	65.28 ± 2.43
Autumn White	34.54 ± 1.28	122.61 ± 3.39	398.76 ± 16.76	26.21 ± 0.74	35.40 ± 1.43	81.81 ± 2.22	417.70 ± 24.85	96.00 ± 1.38	55.28 ± 2.54	168.69 ± 4.82	33.08 ± 0.74	878.96 ± 29.43	152.33 ± 5.49	68.42 ± 2.48
Baibigebuer	25.43 ± 1.04	109.48 ± 2.65	452.49 ± 17.58	17.87 ± 0.64	42.32 ± 2.14	68.29 ± 2.31	423.76 ± 22.41	88.39 ± 2.38	49.75 ± 2.31	186.39 ± 8.32	44.44 ± 0.48	859.29 ± 33.29	98.28 ± 2.18	58.29 ± 3.01
Baidehai	28.75 ± 1.32	112.38 ± 3.82	410.38 ± 15.82	25.65 ± 1.32	30.26 ± 1.43	76.47 ± 3.18	455.56 ± 18.94	94.81 ± 2.18	68.47 ± 2.43	175.58 ± 5.30	46.39 ± 0.65	943.67 ± 28.65	102.48 ± 3.92	55.34 ± 2.76
Blauer Trollinger	36.38 ± 2.23	139.91 ± .521	732.48 ± 27.93	38.39 ± 2.28	29.44 ± 0.63	80.28 ± 3.87	521.53 ± 16.84	67.29 ± 1.38	84.28 ± 2.11	159.44 ± 6.06	29.54 ± 0.48	848.27 ± 33.18	168.39 ± 6.43	70.66 ± 3.23
Blue French	40.11 ± 1.34	126.53 ± 2.31	538.81 ± 15.66	48.32 ± 2.37	53.24 ± 2.39	71.81 ± 0.89	582.42 ± 20.89	80.22 ± 1.88	68.40 ± 1.25	178.92 ± 4.49	18.57 ± 1.73	1082.99 ± 35.04	177.51 ± 5.18	77.76 ± 2.67
Bourboulenc	34.82 ± 0.88	119.53 ± 1.32	427.13 ± 14.47	24.35 ± 1.38	38.53 ± 0.87	88.73 ± 5.53	369.21 ± 14.65	71.76 ± 1.68	56.22 ± 2.71	171.90 ± 3.53	42.91 ± 1.96	981.86 ± 29.84	98.36 ± 3.48	67.92 ± 2.23
Cabernet Franc	23.03 ± 0.78	126.47 ± 2.35	436.61 ± 18.49	47.56 ± 2.82	50.48 ± 1.38	84.38 ± 3.12	615.81 ± 33.79	79.37 ± 2.22	84.75 ± 2.12	169.28 ± 3.89	25.67 ± 0.78	1103.57 ± 49.93	183.94 ± 6.40	83.56 ± 4.06
Cabernet Gernischt	24.53 ± 0.78	132.10 ± 2.44	736.22 ± 26.25	40.26 ± 1.79	43.65 ± 0.85	86.28 ± 2.73	449.44 ± 34.75	75.31 ± 1.85	81.30 ± 0.95	168.59 ± 4.88	21.64 ± 0.92	973.36 ± 23.88	173.42 ± 7.58	77.89 ± 1.93
Cabernet Sauvignon	42.94 ± 1.38	142.01 ± 2.31	741.35 ± 26.46	43.81 ± 1.82	45.38 ± 1.38	110.96 ± 0.72	547.91 ± 23.39	79.78 ± 2.22	90.46 ± 4.40	176.08 ± 2.47	26.28 ± 0.82	1038.55 ± 35.31	194.82 ± 3.38	88.05 ± 3.18
Carignane	34.89 ± 0.98	117.75 ± 4.43	729.27 ± 25.76	34.28 ± 0.91	55.23 ± 2.43	84.08 ± 5.88	417.33 ± 14.93	74.47 ± 1.38	55.84 ± 1.82	169.91 ± 6.21	23.75 ± 1.44	732.62 ± 20.63	160.42 ± 6.75	72.73 ± 1.92
Centennial Seedless	26.47 ± 1.20	98.45 ± 3.56	439.29 ± 16.92	22.37 ± 0.73	36.58 ± 1.48	75.38 ± 3.19	398.87 ± 12.56	90.28 ± 2.68	69.29 ± 2.05	194.58 ± 6.38	37.19 ± 2.15	873.19 ± 22.34	69.32 ± 2.19	63.29 ± 2.43
Chardonnay	15.57 ± 1.42	108.08 ± 1.21	489.66 ± 13.46	19.44 ± 0.93	66.48 ± 2.84	94.83 ± 2.72	406.85 ± 21.34	85.90 ± 2.51	53.50 ± 1.30	170.40 ± 4.98	51.77 ± 0.88	826.88 ± 25.43	130.47 ± 5.31	67.88 ± 2.08
Chenin Blanc	57.21 ± 1.83	126.05 ± 4.32	395.75 ± 15.47	23.18 ± 1.30	39.33 ± 2.05	78.39 ± 3.78	391.11 ± 6.73	97.28 ± 2.88	62.01 ± 1.84	198.71 ± 4.63	46.99 ± 0.88	765.29 ± 25.59	121.33 ± 4.53	60.38 ± 2.76

Table S1. *Cont.*

Variety (*V. vinifera L*)	Gallic acid	Protocatechuic acid	Catechin	Vanillic acid	Caffeic acid	Syringic acid	Epicatechin	*p*-Coumaric acid	Rutin	Salicylic acid	Coumarin	*trans*-Resveratrol	Morin	Quercetin
Cinsaut	37.22 ± 0.98	111.12 ± 3.31	526.03 ± 15.56	45.28 ± 1.98	56.23 ± 2.54	92.26 ± 4.69	544.86 ± 22.94	88.88 ± 2.55	67.02 ± 2.79	176.68 ± 4.20	22.77 ± 0.93	922.32 ± 22.79	148.50 ± 3.65	81.76 ± 0.82
Colombard	34.58 ± 0.85	103.44 ± 4.28	469.29 ± 16.38	34.92 ± 2.03	41.38 ± 1.55	77.23 ± 3.10	438.39 ± 21.23	108.38 ± 4.21	61.28 ± 2.30	204.39 ± 9.48	43.19 ± 1.50	749.48 ± 33.18	143.39 ± 4.28	56.49 ± 1.38
Crimson Seedless	29.08 ± 0.68	115.47 ± 3.28	531.03 ± 14.29	30.38 ± 0.94	38.54 ± 1.43	83.48 ± 2.82	453.94 ± 18.23	85.29 ± 3.11	71.49 ± 3.28	169.39 ± 5.35	19.83 ± 0.67	759.35 ± 35.10	118.54 ± 3.21	58.29 ± 3.04
Ecoly	21.44 ± 0.98	124.63 ± 2.31	453.68 ± 15.67	38.24 ± 2.31	40.23 ± 2.04	80.20 ± 2.69	370.28 ± 13.85	94.21 ± 1.23	51.70 ± 0.78	171.46 ± 1.92	33.63 ± 1.22	663.77 ± 19.87	105.69 ± 5.42	67.74 ± 1.16
Fenghuang 51	17.54 ± 0.63	107.20 ± 2.08	479.64 ± 15.63	41.32 ± 1.59	44.25 ± 1.59	79.02 ± 2.31	477.71 ± 32.12	70.55 ± 1.88	67.79 ± 1.95	168.76 ± 3.84	35.27 ± 0.83	1350.91 ± 29.93	134.48 ± 5.23	68.28 ± 2.38
Flamming Muscat	40.22 ± 1.88	122.15 ± 2.43	473.66 ± 14.63	38.41 ± 2.11	36.34 ± 1.49	88.16 ± 5.45	405.21 ± 31.53	72.80 ± 1.82	53.33 ± 1.74	168.45 ± 5.76	28.83 ± 0.57	749.86 ± 15.65	140.35 ± 7.27	69.73 ± 2.32
Gamay	36.28 ± 0.22	119.75 ± 4.18	512.79 ± 12.34	40.23 ± 2.81	57.38 ± 1.46	72.49 ± 5.52	372.01 ± 11.99	71.58 ± 1.65	73.83 ± 1.92	171.01 ± 3.42	25.56 ± 0.77	960.50 ± 21.98	161.19 ± 5.94	69.85 ± 3.45
Gamay Blanc	16.53 ± 0.52	127.27 ± 2.18	462.57 ± 15.78	27.54 ± 1.67	50.47 ± 1.39	86.47 ± 5.22	337.01 ± 8.77	72.05 ± 2.75	56.61 ± 0.68	183.45 ± 5.48	54.77 ± 1.35	856.53 ± 23.54	139.48 ± 7.55	68.14 ± 3.61
Gamay Noir	52.45 ± 1.52	151.56 ± 5.88	783.21 ± 29.95	36.36 ± 1.49	35.43 ± 0.78	93.76 ± 3.42	656.67 ± 22.76	86.04 ± 3.42	70.77 ± 1.32	173.76 ± 5.78	36.14 ± 1.12	1290.95 ± 93.48	163.85 ± 6.98	71.77 ± 2.68
Gewürztraminer	30.11 ± 1.93	105.61 ± 3.69	453.65 ± 12.67	39.28 ± 2.02	40.35 ± 1.79	74.58 ± 2.48	425.48 ± 17.77	77.93 ± 1.85	56.45 ± 2.72	176.16 ± 5.39	23.34 ± 0.92	825.03 ± 33.64	120.48 ± 4.62	69.04 ± 5.43
Gouais Blanc	26.26 ± 0.58	110.65 ± 2.43	416.03 ± 12.73	19.22 ± 2.10	38.28 ± 1.55	91.21 ± 2.66	387.28 ± 14.55	82.82 ± 1.88	52.52 ± 1.52	174.51 ± 4.83	31.95 ± 1.27	700.11 ± 29.03	139.50 ± 4.43	57.85 ± 1.20
Granoir	43.45 ± 1.58	128.90 ± 2.33	514.01 ± 16.33	35.21 ± 1.32	42.58 ± 1.84	87.94 ± 2.73	494.24 ± 25.83	77.89 ± 0.92	78.46 ± 1.86	175.68 ± 3.55	22.62 ± 0.48	852.19 ± 23.83	138.55 ± 3.41	68.63 ± 2.55
Grenache Blanc	15.79 ± 0.28	115.04 ± 2.31	356.77 ± 12.43	28.23 ± 1.52	40.22 ± 1.68	78.36 ± 0.74	435.64 ± 30.79	96.32 ± 1.52	53.22 ± 1.96	198.48 ± 4.83	30.84 ± 0.59	828.19 ± 26.32	135.84 ± 6.42	67.72 ± 1.98
Guibao	44.38 ± 0.89	129.59 ± 3.24	467.27 ± 16.44	35.30 ± 2.33	39.84 ± 1.93	100.79 ± 4.89	373.78 ± 10.42	74.65 ± 1.38	63.05 ± 0.92	171.67 ± 1.18	28.98 ± 1.21	761.11 ± 17.48	145.48 ± 6.48	78.77 ± 2.04
Heijixin	12.81 ± 0.16	112.42 ± 2.27	529.42 ± 13.46	38.43 ± 1.28	48.75 ± 2.11	71.89 ± 4.52	540.29 ± 29.51	72.60 ± 1.56	60.74 ± 2.54	169.11 ± 2.87	29.62 ± 1.65	676.23 ± 22.45	153.33 ± 7.43	69.88 ± 3.39
Itlian Riesling	40.49 ± 1.53	109.25 ± 5.38	441.47 ± 14.43	33.67 ± 2.31	60.38 ± 2.34	72.24 ± 1.82	392.83 ± 26.76	105.46 ± 1.78	55.68 ± 2.13	201.68 ± 6.02	28.07 ± 0.78	728.83 ± 17.39	112.37 ± 4.73	70.22 ± 1.93
Jasmin	31.93 ± 0.32	112.74 ± 2.32	468.45 ± 15.73	45.32 ± 2.88	42.48 ± 2.48	75.92 ± 2.88	481.22 ± 19.72	70.31 ± 0.93	72.05 ± 1.42	169.68 ± 4.69	32.50 ± 3.45	918.00 ± 38.41	132.56 ± 5.83	68.83 ± 0.99
Jingxiu	27.14 ± 0.88	128.68 ± 2.36	558.77 ± 15.75	36.39 ± 1.97	41.39 ± 1.58	72.06 ± 2.79	510.14 ± 31.74	71.34 ± 1.84	69.17 ± 2.34	170.37 ± 4.21	28.00 ± 0.80	631.76 ± 23.29	140.35 ± 6.83	68.14 ± 1.55
Jingyu	54.29 ± 1.98	134.37 ± 4.13	409.58 ± 15.63	30.91 ± 1.44	58.54 ± 3.03	86.01 ± 2.76	443.69 ± 23.82	85.05 ± 2.33	74.39 ± 3.58	171.46 ± 6.49	29.91 ± 0.68	997.62 ± 42.42	132.42 ± 5.64	59.06 ± 2.89
Jiubai	58.8 ± 2.38	126.52 ± 2.30	497.93 ± 17.53	28.36 ± 0.38	50.24 ± 2.33	87.52 ± 3.52	487.59 ± 41.32	88.62 ± 1.79	68.44 ± 3.32	171.79 ± 4.56	33.00 ± 0.92	570.75 ± 22.54	114.76 ± 4.77	57.97 ± 2.22
Lungyen	32.32 ± 1.32	135.44 ± 2.34	484.67 ± 16.64	33.83 ± 1.34	48.56 ± 2.37	83.28 ± 5.53	556.16 ± 25.88	79.33 ± 2.14	50.93 ± 1.74	168.24 ± 5.53	27.69 ± 1.85	744.64 ± 21.58	123.37 ± 5.73	67.84 ± 2.88
Manaizi	14.67 ± 0.06	116.69 ± 2.33	453.59 ± 16.32	28.57 ± 1.48	63.25 ± 3.13	83.73 ± 4.63	377.58 ± 22.32	81.09 ± 1.75	63.24 ± 1.98	170.76 ± 5.83	44.32 ± 0.84	693.25 ± 27.46	144.85 ± 7.02	68.52 ± 1.92
Manicure Finger	30.91 ± 1.18	124.44 ± 3.35	707.72 ± 27.49	31.24 ± 1.17	50.89 ± 2.38	73.62 ± 2.54	384.61 ± 13.42	71.69 ± 1.09	55.41 ± 2.32	178.28 ± 4.44	24.72 ± 0.59	1074.03 ± 32.83	132.83 ± 5.84	67.81 ± 1.82
Merlot	14.53 ± 0.89	157.73 ± 3.34	839.69 ± 29.74	48.31 ± 2.57	43.28 ± 2.08	74.59 ± 4.71	540.13 ± 16.83	102.57 ± 2.39	70.63 ± 2.85	168.97 ± 4.36	28.05 ± 2.48	848.58 ± 24.49	176.43 ± 8.28	67.97 ± 2.69
Mission	26.19 ± 0.62	122.91 ± 4.42	589.94 ± 13.46	44.75 ± 2.55	45.25 ± 1.78	73.30 ± 2.12	656.80 ± 35.54	75.20 ± 1.02	83.34 ± 1.79	174.63 ± 5.56	24.11 ± 0.88	840.74 ± 25.21	167.69 ± 6.45	70.65 ± 3.21

Table S1. *Cont.*

Variety (*V. vinifera L*)	Gallic acid	Protocatechuic acid	Catechin	Vanillic acid	Caffeic acid	Syringic acid	Epicatechin	*p*-Coumaric acid	Rutin	Salicylic acid	Coumarin	*trans*-Resveratrol	Morin	Quercetin
Monukka	24.48 ± 1.20	137.58 ± 4.32	649.28 ± 16.92	38.67 ± 1.38	48.48 ± 2.18	84.65 ± 3.84	548.20 ± 20.19	72.18 ± 2.33	68.98 ± 2.66	153.58 ± 4.55	26.38 ± 0.85	698.39 ± 27.33	155.65 ± 4.78	68.94 ± 2.83
Müller-Thurgau	20.31 ± 0.89	167.97 ± 4.31	401.52 ± 15.82	31.73 ± 2.13	65.20 ± 3.32	103.59 ± 6.53	463.75 ± 21.83	131.67 ± 2.98	84.22 ± 7.73	227.96 ± 2.42	45.39 ± 0.87	664.66 ± 23.44	143.41 ± 5.32	67.96 ± 1.38
Munage	37.84 ± 1.12	131.38 ± 5.51	589.92 ± 13.32	38.56 ± 0.57	50.26 ± 2.87	76.24 ± 3.52	446.95 ± 13.75	85.38 ± 2.24	83.18 ± 2.86	175.64 ± 2.98	33.63 ± 1.08	615.57 ± 27.41	131.01 ± 5.57	69.37 ± 4.10
Muscat Blanc	19.12 ± 0.78	128.00 ± 4.32	465.90 ± 16.48	40.36 ± 1.04	66.39 ± 3.33	91.78 ± 2.42	469.17 ± 26.74	95.98 ± 1.52	69.07 ± 2.31	199.31 ± 3.62	38.88 ± 1.04	1047.44 ± 32.19	113.38 ± 4.89	57.87 ± 2.83
Muscat Hamburg	36.39 ± 0.52	129.91 ± 2.31	620.61 ± 17.46	42.39 ± 2.31	39.67 ± 1.85	138.32 ± 7.59	656.98 ± 27.89	85.58 ± 1.89	73.18 ± 1.65	168.92 ± 2.44	29.36 ± 1.13	1093.25 ± 45.59	167.91 ± 6.08	68.35 ± 2.52
Muscat Mathiasz Janosne	25.32 ± 0.98	127.85 ± 2.72	543.22 ± 15.77	36.19 ± 1.43	47.29 ± 2.54	85.43 ± 3.85	385.40 ± 19.73	75.38 ± 1.43	52.33 ± 1.82	168.95 ± 5.48	21.70 ± 1.02	1016.91 ± 48.83	142.36 ± 4.59	67.82 ± 2.30
Muscat of Alexandria	40.59 ± 2.08	125.07 ± 5.44	427.49 ± 13.87	40.81 ± 1.77	59.49 ± 1.55	87.96 ± 1.77	382.36 ± 16.78	84.49 ± 1.12	55.18 ± 0.69	181.22 ± 3.33	41.58 ± 1.09	908.93 ± 22.29	123.59 ± 6.44	62.55 ± 1.22
Nebbiolo	21.31 ± 0.48	125.27 ± 3.35	516.06 ± 18.52	44.29 ± 2.19	39.83 ± 0.83	86.54 ± 6.12	533.60 ± 30.78	78.58 ± 0.92	66.83 ± 0.88	170.41 ± 3.88	32.74 ± 0.98	898.91 ± 38.72	188.33 ± 5.28	68.58 ± 2.68
Niunai	30.78 ± 2.33	123.03 ± 2.54	428.49 ± 16.29	28.39 ± 1.93	53.69 ± 2.65	78.58 ± 2.32	398.28 ± 25.39	79.28 ± 2.32	65.58 ± 1.39	189.39 ± 5.39	29.54 ± 0.65	793.67 ± 22.48	109.50 ± 3.21	52.49 ± 1.66
Pearl of Csaba	19.39 ± 0.24	127.38 ± 2.31	428.64 ± 15.63	34.83 ± 2.14	66.45 ± 2.38	67.72 ± 2.85	380.43 ± 25.78	84.90 ± 2.44	59.45 ± 2.58	195.46 ± 3.57	28.05 ± 0.98	882.38 ± 21.29	120.36 ± 4.40	54.43 ± 2.75
Petit Manseng	34.84 ± 1.32	125.84 ± 4.32	357.66 ± 14.71	24.47 ± 0.73	59.29 ± 2.15	76.98 ± 4.02	367.10 ± 42.57	75.23 ± 1.33	58.89 ± 1.59	179.59 ± 4.59	29.80 ± 0.77	937.79 ± 27.47	122.48 ± 5.37	49.27 ± 3.54
Petit Verdot	33.38 ± 0.92	118.16 ± 5.38	629.96 ± 13.66	39.31 ± 1.32	41.32 ± 1.09	98.12 ± 5.41	452.08 ± 13.74	80.34 ± 4.72	73.85 ± 5.48	173.45 ± 6.39	19.77 ± 0.38	1288.13 ± 45.93	103.39 ± 4.63	79.88 ± 3.90
Pinot Blanc	19.37 ± 0.82	125.57 ± 1.34	345.32 ± 16.61	37.26 ± 0.88	63.32 ± 2.69	84.65 ± 4.75	410.93 ± 34.51	85.19 ± 2.20	60.43 ± 2.44	168.53 ± 4.53	36.65 ± 1.08	1192.34 ± 22.12	105.36 ± 5.15	61.76 ± 3.47
Pinot Gris	21.59 ± 0.33	127.32 ± 3.32	566.56 ± 24.76	41.30 ± 2.37	45.25 ± 1.94	90.41 ± 2.52	447.75 ± 12.42	76.75 ± 1.88	54.48 ± 1.66	170.55 ± 1.93	20.81 ± 0.04	1129.93 ± 13.89	169.40 ± 5.49	68.11 ± 3.32
Pinot Noir	19.39 ± 0.21	124.60 ± 1.78	565.79 ± 21.41	48.28 ± 2.75	42.10 ± 2.11	75.08 ± 4.54	459.28 ± 16.88	72.69 ± 1.86	67.41 ± 1.89	171.28 ± 5.58	19.62 ± 1.25	1551.55 ± 62.49	170.68 ± 6.59	77.81 ± 2.73
Pollux	24.77 ± 0.21	125.53 ± 2.31	412.89 ± 18.47	36.21 ± 2.31	57.22 ± 1.89	90.69 ± 4.51	480.22 ± 14.75	76.89 ± 1.59	54.66 ± 1.74	182.26 ± 2.43	30.71 ± 1.28	829.19 ± 13.44	105.47 ± 4.56	67.75 ± 4.49
Queen of Vineyard	28.97 ± 1.38	126.09 ± 2.33	427.37 ± 17.26	38.92 ± 2.47	60.21 ± 2.18	110.65 ± 2.29	431.04 ± 35.97	106.00 ± 2.79	85.92 ± 2.84	174.59 ± 4.92	23.26 ± 0.82	771.24 ± 15.43	132.47 ± 5.93	59.30 ± 3.13
Red Globe	39.09 ± 3.43	133.37 ± 2.98	540.29 ± 14.98	42.48 ± 2.67	44.73 ± 2.54	84.57 ± 3.48	482.38 ± 32.03	81.39 ± 3.41	68.95 ± 2.39	167.39 ± 5.82	25.49 ± 1.58	955.29 ± 30.39	168.52 ± 4.28	58.29 ± 2.55
Red Guibao	55.59 ± 1.78	118.04 ± 1.38	574.60 ± 14.42	39.04 ± 1.54	48.67 ± 2.13	76.05 ± 3.89	359.81 ± 20.32	74.33 ± 1.65	90.76 ± 3.76	178.55 ± 5.44	23.79 ± 1.22	992.45 ± 31.54	182.53 ± 6.59	69.93 ± 1.59
Riesling	31.26 ± 1.34	110.46 ± 2.65	430.86 ± 13.45	35.87 ± 2.13	58.28 ± 1.94	89.32 ± 4.64	397.39 ± 33.42	97.62 ± 1.58	61.02 ± 2.35	199.04 ± 4.93	39.75 ± 1.16	1072.17 ± 34.52	125.41 ± 4.59	58.17 ± 1.85
Rizamat	13.52 ± 0.78	112.32 ± 2.39	399.61 ± 11.41	30.84 ± 0.88	51.47 ± 2.19	81.08 ± 0.56	500.43 ± 39.43	71.56 ± 1.62	52.92 ± 1.52	168.87 ± 4.29	26.74 ± 0.84	703.44 ± 19.94	105.39 ± 5.32	62.12 ± 1.89
Rkatsiteli	17.88 ± 0.55	133.04 ± 2.32	492.61 ± 13.43	37.38 ± 1.05	63.31 ± 3.01	80.30 ± 6.55	451.37 ± 19.85	90.79 ± 1.32	56.80 ± 1.73	187.77 ± 4.72	43.40 ± 1.52	870.63 ± 26.92	112.55 ± 4.59	45.73 ± 3.75

Table S1. *Cont.*

Variety (*V. vinifera L*)	Gallic acid	Protocatechuic acid	Catechin	Vanillic acid	Caffeic acid	Syringic acid	Epicatechin	*p*-Coumaric acid	Rutin	Salicylic acid	Coumarin	*trans*-Resveratrol	Morin	Quercetin
Roussanne Duvar	22.34 ± 0.78	131.41 ± 4.31	600.44 ± 17.64	43.23 ± 3.12	55.63 ± 2.50	75.02 ± 2.89	523.00 ± 18.98	89.96 ± 2.34	52.96 ± 1.83	172.06 ± 4.69	23.31 ± 0.86	1154.51 ± 27.82	102.42 ± 3.58	68.23 ± 1.87
Ruby Cabernet	41.38 ± 2.89	137.92 ± 3.64	596.28 ± 18.29	46.92 ± 1.89	39.96 ± 1.49	87.48 ± 3.18	502.48 ± 21.39	79.29 ± 3.51	58.43 ± 1.86	180.38 ± 6.83	38.28 ± 1.55	983.45 ± 33.29	138.39 ± 4.29	70.39 ± 3.29
Ruby Seedless	47.65 ± 2.38	141.61 ± 5.36	514.28 ± 16.48	41.43 ± 1.75	53.86 ± 2.39	80.89 ± 5.57	459.93 ± 23.83	70.74 ± 1.88	56.55 ± 1.46	170.22 ± 4.84	23.45 ± 0.85	886.12 ± 24.822	187.36 ± 8.94	78.30 ± 4.02
Sangiovese	25.83 ± 0.58	142.84 ± 5.33	694.45 ± 13.58	48.28 ± 2.29	50.46 ± 1.86	83.84 ± 3.69	545.29 ± 35.58	72.91 ± 2.03	60.21 ± 2.55	169.25 ± 3.98	25.04 ± 1.73	850.53 ± 18.74	174.34 ± 6.54	69.14 ± 2.96
Saperavi	21.03 ± 1.04	117.72 ± 1.52	552.86 ± 17.65	46.84 ± 1.73	55.29 ± 2.14	80.72 ± 2.72	515.38 ± 25.70	70.73 ± 1.99	86.00 ± 4.79	169.45 ± 5.22	27.80 ± 1.40	780.86 ± 31.63	179.73 ± 5.42	70.04 ± 1.92
Sauvignon Blanc	17.23 ± 0.18	111.47 ± 2.31	545.60 ± 14.89	29.38 ± 1.38	71.30 ± 3.10	95.77 ± 2.79	418.09 ± 13.98	74.94 ± 1.67	52.94 ± 0.82	181.52 ± 2.68	38.93 ± 1.24	899.88 ± 19.98	134.51 ± 5.44	67.74 ± 3.08
Seibel Noir	41.36 ± 1.78	119.11 ± 1.36	658.23 ± 16.42	45.34 ± 2.44	58.32 ± 1.78	84.21 ± 3.62	467.78 ± 32.18	72.40 ± 1.26	64.18 ± 2.44	170.25 ± 4.12	21.93 ± 0.69	837.91 ± 34.31	176.59 ± 6.76	74.05 ± 2.54
Semillon	21.94 ± 0.58	135.69 ± 0.48	583.49 ± 21.44	27.38 ± 0.89	65.38 ± 2.32	100.67 ± 5.88	470.11 ± 23.45	89.63 ± 2.45	53.99 ± 1.61	169.75 ± 1.43	35.94 ± 0.75	1090.83 ± 12.42	142.32 ± 6.78	67.74 ± 1.96
Shandongzaohong	37.75 ± 1.43	122.03 ± 3.35	420.23 ± 14.42	38.31 ± 1.88	59.28 ± 3.02	94.55 ± 4.75	411.89 ± 20.48	73.78 ± 2.34	60.67 ± 0.72	172.19 ± 3.78	15.03 ± 1.05	941.76 ± 27.83	154.54 ± 4.92	78.88 ± 3.23
Silvaner	30.32 ± 2.14	123.94 ± 2.17	482.58 ± 15.92	37.19 ± 2.14	72.13 ± 2.89	77.39 ± 2.85	589.29 ± 14.93	88.35 ± 1.28	64.76 ± 2.70	184.38 ± 5.38	34.66 ± 0.89	1027.38 ± 33.28	105.34 ± 2.57	50.32 ± 2.56
Syrah	29.61 ± 0.08	126.53 ± 1.39	451.46 ± 16.11	46.32 ± 2.54	66.32 ± 2.53	88.96 ± 1.78	413.81 ± 18.75	75.73 ± 1.87	67.67 ± 1.75	172.21 ± 3.82	29.91 ± 0.66	897.11 ± 16.83	158.47 ± 4.83	83.78 ± 3.11
Thompson Seedless	21.48 ± 0.98	91.67 ± 2.24	344.69 ± 12.48	24.19 ± 0.58	65.36 ± 1.85	71.73 ± 1.42	450.36 ± 18.72	79.81 ± 1.62	50.39 ± 0.82	177.65 ± 5.58	28.56 ± 2.02	771.87 ± 10.31	133.27 ± 4.94	67.71 ± 4.05
Ugni Blanc	11.54 ± 1.42	124.33 ± 2.39	530.27 ± 13.76	33.38 ± 1.42	70.25 ± 2.41	87.51 ± 3.88	426.42 ± 20.38	101.97 ± 1.76	62.36 ± 2.63	198.41 ± 5.42	32.77 ± 1.28	801.50 ± 15.74	131.22 ± 5.48	61.88 ± 2.43
Victoria Blanc	29.25 ± 0.99	127.35 ± 1.28	575.74 ± 23.46	30.31 ± 0.88	68.82 ± 2.56	82.54 ± 1.92	410.08 ± 33.80	73.30 ± 0.98	62.78 ± 3.02	202.30 ± 5.33	20.13 ± 1.86	704.49 ± 25.32	122.33 ± 4.89	68.54 ± 1.86
Wuyuezi	44.54 ± 2.28	145.53 ± 3.45	776.14 ± 22.01	41.39 ± 3.04	49.93 ± 2.17	113.37 ± 4.54	646.65 ± 33.54	72.92 ± 1.76	54.67 ± 2.22	170.49 ± 3.77	25.13 ± 1.29	772.74 ± 32.89	178.51 ± 7.81	59.76 ± 2.03
Xiabai	20.02 ± 0.93	127.35 ± 5.42	389.84 ± 12.51	25.45 ± 0.76	68.35 ± 2.11	81.85 ± 4.76	484.65 ± 28.62	76.89 ± 2.02	63.97 ± 2.83	168.68 ± 3.37	33.11 ± 1.49	686.03 ± 24.53	130.55 ± 4.84	55.72 ± 3.12
Yan73	20.73 ± 3.38	119.88 ± 3.41	560.09 ± 15.55	41.30 ± 2.86	55.27 ± 2.52	82.17 ± 2.33	468.30 ± 22.02	77.75 ± 2.43	61.52 ± 2.29	160.84 ± 3.84	36.58 ± 1.43	628.75 ± 28.40	200.53 ± 7.43	69.42 ± 2.04
Yan74	15.89 ± 0.68	118.83 ± 1.38	536.38 ± 12.46	44.37 ± 3.11	60.39 ± 3.01	80.02 ± 4.68	461.99 ± 22.82	71.00 ± 1.42	55.93 ± 2.62	168.63 ± 5.02	39.67 ± 0.87	613.27 ± 20.82	195.34 ± 7.55	65.15 ± 2.84
Zaobai	16.21 ± 0.48	109.40 ± 2.12	306.86 ± 15.74	28.83 ± 0.83	79.48 ± 3.19	82.27 ± 2.40	413.14 ± 24.43	71.49 ± 1.77	55.01 ± 1.92	177.74 ± 4.29	29.58 ± 0.54	834.63 ± 28.32	142.84 ± 5.44	53.75 ± 2.37
Zexiang	16.08 ± 0.08	107.48 ± 1.25	406.80 ± 12.78	30.91 ± 1.32	77.29 ± 2.87	88.36 ± 4.73	368.25 ± 21.22	71.80 ± 1.79	58.60 ± 2.23	180.13 ± 4.18	32.80 ± 2.11	891.58 ± 32.45	138.39 ± 5.13	63.78 ± 3.91
Zinfandel	20.92 ± 0.38	123.68 ± 2.35	721.89 ± 18.47	45.29 ± 3.11	59.49 ± 1.98	88.29 ± 6.87	597.51 ± 23.57	79.25 ± 2.44	64.84 ± 1.46	171.05 ± 2.56	30.92 ± 1.44	706.57 ± 35.52	156.38 ± 5.99	69.74 ± 3.32
Клерет белый (Clairette Doree)	15.82 ± 1.23	124.79 ± 5.38	471.18 ± 13.48	35.82 ± 2.48	85.54 ± 3.19	95.58 ± 4.77	393.21 ± 13.40	77.90 ± 1.39	60.23 ± 2.34	175.97 ± 3.78	29.87 ± 0.88	892.40 ± 22.94	122.37 ± 3.66	69.57 ± 2.35
